# A thiol-reactive Ru(II) ion, not CO release, underlies the potent antimicrobial and cytotoxic properties of CO-releasing molecule-3

**DOI:** 10.1016/j.redox.2018.06.008

**Published:** 2018-06-30

**Authors:** Hannah M. Southam, Thomas W. Smith, Rhiannon L. Lyon, Chunyan Liao, Clare R. Trevitt, Laurence A. Middlemiss, Francesca L. Cox, Jonathan A. Chapman, Sherif F. El-Khamisy, Michael Hippler, Michael P. Williamson, Peter J.F. Henderson, Robert K. Poole

**Affiliations:** a Department of Molecular Biology and Biotechnology, The University of Sheffield, Western Bank, Sheffield S10 2TN, UK; b Department of Chemistry, The University of Sheffield, Western Bank, Sheffield S3 7HF, UK; c School of Biomedical Sciences and Astbury Centre for Structural Molecular Biology, University of Leeds, Leeds LS2 9JT, UK

**Keywords:** CORM-3, Ru(CO)_3_Cl(glycinate), DMEM, Dulbecco's Modified Eagle Medium, FCS, fetal calf serum, FTIR, Fourier-transform infrared spectroscopy, GDMM, glucose defined minimal medium, GSH, glutathione, GSSG, glutathione disulfide, ICP-AES, inductively coupled plasma-atomic emission spectroscopy, KPi, inorganic phosphate buffer, LB, lysogeny broth, Mb, myoglobin, MH-II, Mueller-Hinton medium (II), MIC, minimal inhibitory concentration, NAC, *N*-acetyl cysteine, PBS, phosphate-buffered saline, Red-Mb, reduced myoglobin, RPMI, Roswell Park Memorial Institute Medium, WGSR, water-gas shift reaction, Novel antimicrobials, CO-releasing molecules, Gasotransmitters, CORM-3, Metallo-drugs

## Abstract

Carbon monoxide (CO)-releasing molecules (CORMs), mostly metal carbonyl compounds, are extensively used as experimental tools to deliver CO, a biological ‘gasotransmitter’, in mammalian systems. CORMs are also explored as potential novel antimicrobial drugs, effectively and rapidly killing bacteria in vitro and in animal models, but are reportedly benign towards mammalian cells. Ru-carbonyl CORMs, exemplified by CORM-3 (Ru(CO)_3_Cl(glycinate)), exhibit the most potent antimicrobial effects against *Escherichia coli*. We demonstrate that CORM-3 releases little CO in buffers and cell culture media and that the active antimicrobial agent is Ru(II), which binds tightly to thiols. Thus, thiols and amino acids in complex growth media – such as histidine, methionine and oxidised glutathione, but most pertinently cysteine and reduced glutathione (GSH) – protect both bacterial and mammalian cells against CORM-3 by binding and sequestering Ru(II). No other amino acids exert significant protective effects. NMR reveals that CORM-3 binds cysteine and GSH in a 1:1 stoichiometry with dissociation constants, K_d_, of about 5 μM, while histidine, GSSG and methionine are bound less tightly, with K_d_ values ranging between 800 and 9000 μM. There is a direct positive correlation between protection and amino acid affinity for CORM-3. Intracellular targets of CORM-3 in both bacterial and mammalian cells are therefore expected to include GSH, free Cys, His and Met residues and any molecules that contain these surface-exposed amino acids. These results necessitate a major reappraisal of the biological effects of CORM-3 and related CORMs.

## Highlights

• Carbon monoxide-releasing molecules (CORMs) are used for experimental CO delivery.
• CORM-3 is a potent antimicrobial, but is reportedly beneficial to mammalian cells.
• We demonstrate CORM-3 releases little CO in buffers and cell culture media.
• Redox-active Ru^2+^ is the biological agent, binding tightly to metabolites e.g. thiol.
• These results necessitate a major reappraisal of the biological effects of CORMs.

## Graphical abstract

**Figure:**
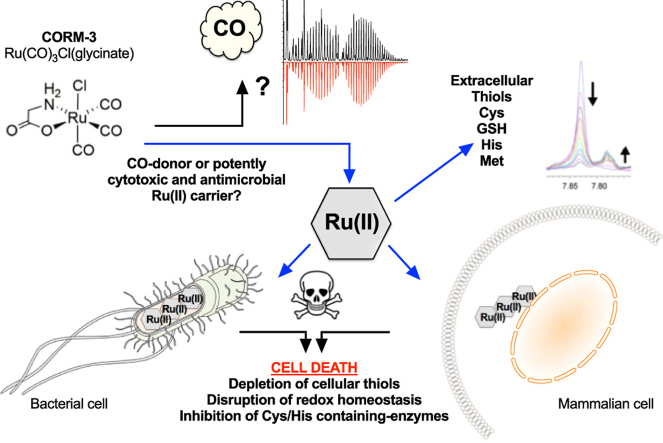

## 1 Introduction

Over the past decade, novel transition metal-based carbon monoxide-releasing molecules (CORMs) have been developed to deliver physiologically relevant levels of CO experimentally or therapeutically [1], [2]. The widespread use of these compounds as CO-donors in over 300 biological studies has accelerated our understanding of CO as an important gasotransmitter molecule in human and animal biology [3]. Increasingly, CORMs are considered as potential pro-drugs for CO delivery, not only in clinical and physiological applications [4], [5], but also as anticancer drugs [6] and antimicrobials [7].

**Fig. 1:**
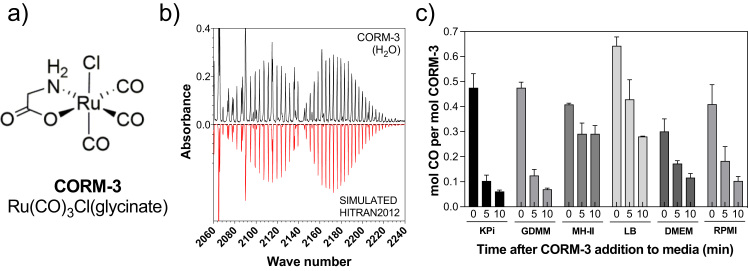
(a) Structure of CORM-3 and (b-c) CORM-3 is an inefficient CO-releasing molecule in commonly used biological media and phosphate buffers. (b) Gas-phase FTIR spectrum of CO released from CORM-3 (100 μM) in H_2_O 30 min after the addition of sodium dithionite (200 μM) (black) shown for comparison against a simulated FTIR spectrum for CO + H_2_O obtained from HITRAN2012 molecular spectroscopic database (red). (c) Total CO released per mol CORM after addition of sodium dithionite following 0, 5 or 10 min incubation of CORM-3 in 30 mM KPi buffer pH 7.4 or various bacterial (GDMM, MH-II, LB) or mammalian cell culture (DMEM or RPMI) media. (For interpretation of the references to color in this figure legend, the reader is referred to the web version of this article.).

One of the earliest and most widely used CORMs is the water-soluble CORM-3 (Ru(CO)_3_Cl(glycinate) Fig. 1(a). Its biological activities are complicated by an extensive solution chemistry [8], [9], [10]: upon solubilisation in water, [Ru(CO)_2_(CO_2_H)Cl(glycinate)]^-^ isomers are generated by attack of hydroxide ions on CORM-3 – the initial step of Water-Gas-Shift-Reaction (WGSR) chemistry. Various biologically active metal-carbonyl complexes are formed, which may exhibit different reactivities and extents of CO-release [10]. CORM-3 has shown promise as an antimicrobial agent in infection models. CORM-3 not only inhibited growth and respiration of *Pseudomonas aeruginosa* in vitro, but also decreased bacterial counts in the spleen of both immunocompetent and immune-suppressed mice [11]. CORM-3 is toxic against laboratory strains and clinical isolates of *Escherichia coli, Campylobacter jejuni, Lactobacillus lactis, Staphylococcus aureus* and *Salmonella enterica* serovar Typhimurium [10]. Injection of the related CORM-2 (Ru_2_Cl_4_(CO)_6_) into mice increased phagocytosis of bacteria and rescued heme oxygenase-deficient mice from sepsis-induced lethality [12]. Since CORM-3 accumulates in bacterial cells (assessed by Ru uptake) and inhibits respiration [13], [14], an early assumption was that its activity was due to intracellular release of CO, which binds to terminal oxidases, thereby inhibiting respiration. There is ample spectroscopic evidence that the released CO binds ferrous hemes in strongly reducing conditions in the absence of oxygen, but any direct link between respiratory inhibition and bactericidal activity is unproven. Thus, CORM-3 is considered primarily a CO-carrier or ‘Trojan Horse’ [15], [16], [17], delivering a toxic cargo of CO, with the residual Ru ion(s) contributing only a minor role in antimicrobial activity. Other investigators have suggested that antimicrobial activity is due in part to generation of reactive oxygen species, perhaps following respiratory inhibition [18], [19]. An important unresolved issue in the potential application of CORMs as antimicrobial drugs is why CORM-3 possesses potent antimicrobial activity, yet is reportedly non-toxic to mammalian cells, ex vivo and whole-animal models, where it exerts therapeutic (including vasodilatory, anti-inflammatory and cardioprotective) effects [20], [21].

A radically different explanation for the toxic biological activities of these Ru-carbonyl CORMs is that, rather than acting via release of CO, they are sources of Ru(II), which reacts with cellular targets. Indeed, over 200 publications report the antimicrobial activities of various Ru-based compounds that are not CORMs; in some, the Ru ions play a direct functional role, directly coordinating to biological targets [10]. Here, we investigate this hypothesis, using a range of biological and biophysical measures, and conclude that CORM-3 releases very little CO under the conditions generally adopted in biological experiments and that the cellular toxicity of CORM-3 is mainly due to the reactions of Ru(II) with thiols and amino acids. These findings have far-reaching implications for the toxicity and pharmacological development of these agents against both bacterial and mammalian cells, and the future use of CORM-3 and related compounds as inert ‘CO-carrier’ vehicles in biological research.

## 2 Materials and methods

### 2.1 CORM-3, tricarbonylchloro (glycinato)ruthenium(II), C_5_H_4_ClNO_5_Ru

CORM-3 was synthesized from CORM-2 (Sigma-Aldrich), as described previously [20]. Stock solutions were in distilled H_2_O (final concentration 1 – 100 mM), shielded from light and used on the day of preparation. Prior to biological assays, CORM-3 solutions were filter-sterilised through a 0.22 µm filter.

### 2.2 CO release from CORM-3

Liberation of CO from CORM-3 was determined by gas-phase Fourier-transform infrared spectroscopy (FTIR) or via myoglobin (Mb) assays. For FTIR, CO detection was as described previously [22] except that a White multiple-pass absorption cell (providing a total folded path length of 8 m) and a cooled detector (EG & G Optoelectronics J15D14 MCT) were used. CO was quantified by Lorentzian fitting of 6 isolated lines (R3, R5, R6, R8, R9 and R10) and comparison of the line integrals with a simulated spectrum generated using absorption coefficients from the HITRAN 2012 database [23]. Measurements of CO release from CORM-3 in various growth media (defined minimal salts medium supplemented with 20 mM glucose ‘GDMM’ [24], LB (Formedium), MH-II (Sigma-Aldrich), DMEM or RPMI (Sigma-Aldrich) or 30 mM KPi buffer pH 7.4 were conducted as follows. CORM-3 (100 μM) was added to 10 mL vigorously stirred, degassed, sterile media or buffer in a sealed vessel connected to the FTIR absorption cell. A peristaltic pump (flow rate 7 L h^−1^) was used to bring the vessel headspace and the IR absorption cell rapidly to equilibrium by cycling the gas through the system. Sodium dithionite (200 μM) was added either immediately prior to CORM-3 or at time intervals after CORM addition. For detecting CO release in the presence of amino acids, 100 μM CORM-3 was added to 30 mM KPi pH 7.4 containing 200 μM Ala, Asp (sodium salt), Cys (hydrochloride), His (monohydrochloride), Met or sodium dithionite (maximum CO release control). In Mb assays, CO release from CORM-3 exploited conversion of reduced myoglobin (red-Mb) to carbonmonoxy-myoglobin (CO-Mb) in vitro [20], [25] (Fig. S1a).

### 2.3 ^1^H NMR

^1^H NMR experiments were conducted in 30 mM KPi buffer prepared in distilled H_2_O at pH 7.4 then freeze-dried and re-dissolved in deuterated water (D_2_O) prior to experiments. For assessment of CORM-3 structural changes that occur upon dissolution in H_2_O or KPi buffer, CORM-3 stocks were prepared either in H_2_O (+ 10% (v/v) D_2_O) or in 0.4–30 mM KPi buffer after pH adjustment with NaOH/HCl as required. For peptide titrations, CORM-3 stocks were prepared in 30 mM KPi buffer and pH adjusted to 7.4 – 7.7 with NaOH. Concentrations of CORM-3 were quantified by ^1^H NMR using 100 μM trimethylsilylpropionate (TSP) as an internal reference, with a 20 s recycle delay for full relaxation between scans. CORM-3 stocks were stored at 4 °C and typically used within 1–2 h. Synthetic peptides (Genscript) A_3_CA_3_, A_3_DA_3_, A_3_HA_3_, A_3_MA_3_ and A_7_ had N-terminal acetylation and C-terminal amidation. Stock solutions of peptides were prepared in 30 mM KPi buffer pH 7.4. Peptides with low solubility in buffer were dissolved in d^6^-DMSO and further diluted with buffer. A maximum of 10% (v/v) d^6^-DMSO was present during titration experiments. Experiments requiring the presence of DMSO were checked against controls containing equivalent concentrations of DMSO alone. Peptide concentrations were determined by ^1^H NMR relative to the internal standard TSP (100 μM). Titrations with reduced glutathione (GSH, Sigma-Aldrich) or oxidised glutathione (GSSG, Sigma-Aldrich) were conducted as for synthetic peptides. ^1^H NMR experiments were carried out on a Bruker Avance-1 800 MHz spectrometer using pre-saturation for solvent suppression. Baselines were corrected manually before spectral signals were integrated using Bruker software. DOSY experiments were performed as described before [26]. Estimations for CORM-peptide binding affinities, expressed as the dissociation constant K_d_, were determined as described [27], based either on integrated peak intensity (slow exchange conditions) or on chemical shift (fast exchange conditions).

### 2.4 General bacterial methods

Bacteria used were *E. coli* K-12 strain MG1655 (F^-^ lambda^-^ *ilvG, rfb-50, rph-1*) or the glutathione-deficient mutant strain *E. coli* MG1655 *gshA*. Culture growth and viability were determined by standard methods (see Supplementary information (SI)). MICs were assessed by exposing 1 × 10^7^ cells to serial dilutions of CORM-3 in 96-well microtiter plates in either glucose defined minimal medium (GDMM) [24] or MH-II. The MIC was determined as the minimum concentration of CORM-3 required to inhibit *E. coli* growth within 16–18 h.

### 2.5 Effects of media, amino acids or sulfur-containing compounds on CORM-3 antimicrobial effects

To investigate the effects of media on CORM-3 activity, a CORM-3 solution (10 mM) was diluted 10-fold with sterile H_2_O (as standard), 10 mM PBS, or one of the following growth media: GDMM, LB, MH-II, DMEM, RPMI-1640. The resulting CORM/media solutions were then incubated at room temperature for 10 min and added to GDMM cultures (OD_595 nm_ ~ 0.23) at a final concentration of 60 μM CORM-3 and 5% (v/v) media. GDMM was supplemented with 0.25% casamino acids to determine the effect of a mixture of amino acids. For assessing effects of individual amino acids (Cys, His, Met, Asp or Ala) or sulfur compounds (GSH, GSSG, sodium hydrosulfide, *N*-acetyl cysteine (NAC), cystine or sodium hydrosulfide) on CORM-3 antimicrobial activity or Ru accumulation, stocks of 10–40 mM CORM-3 were supplemented with a 2-fold excess of amino acid or the sulfur compounds. The resulting mixtures were incubated at room temperature for 10 min and added to bacterial cultures grown in GDMM to give a final concentration of 60 μM CORM-3 and 120 μM amino acid/sulfur compound. Growth and viability of cells was assessed by standard procedures (see SI).

### 2.6 Measurements of Ru content of whole *E. coli* cells

The Ru content of *E. coli* cell pellets was determined by inductively coupled plasma-atomic emission spectroscopy (ICP-AES) at time intervals after the addition of CORM-3 as described previously [13], [16], [28] (see SI).

### 2.7 RKO clonogenicity measurements of cell survival

Mammalian cell culture experiments were performed using human colon carcinoma cell line RKO (ATCC CRL-2577) (see SI). Toxicity was determined by a modified clonogenic assay [29]. RKO cells, grown to 80–90% confluence, were washed and suspended in PBS to 10^6^ cell mL^−1^. Cells were incubated with CORM-3 (0–500 μM) for 1 h at 37 °C and then transferred to fresh RPMI-1640 medium + supplements and grown for 9 days to permit clonal growth. Clonogenicity (i.e. survival and the subsequent ability to form clones) of RKO cells was determined by the average number of colonies arising from each CORM-treated cell suspension, where one colony is representative of one viable cell after acute exposure to the CORM [29]. The % clonogenicity was determined by enumerating CORM-treated RKO cells and cells not treated with CORM. For assessing the effects of media/supplements on CORM-3 cytotoxicity, assays were performed as described above except that cell suspensions of 5 × 10^5^ cells were treated with 25 μM CORM-3 for 1 h in the presence of PBS, PBS + 10% (v/v) FCS (fetal calf serum), PBS + 2 mM L-Gln, DMEM or RPMI-1640 growth media. Thus, clonogenicity of these cells after exposure to CORM-3 was compared to RKO cells treated in the same medium without CORM-3 (see SI).

### 2.8 Measurements of Ru content of RKO cells

Confluent RKO cells were resuspended either in 10 mM PBS or DMEM to a final concentration of 10^6^ cells mL^−1^. CORM-3 (50 μM) was added and the suspensions incubated for 1 h at 37 °C, before centrifugation at 400×*g* for 5 min; the supernatants were discarded. Cells were washed once in ice-cold PBS, then pellets were digested in nitric acid and analysed by ICP-AES for Ru content. The level of accumulated CORM-derived Ru was estimated by the amount of Ru per RKO cell, assuming 3000 µm^3^ for the volume of a typical mammalian epithelial cell [30], assuming a full recovery of 10^6^ cells mL^−1^ in 10 mL prior to ICP-AES.

## 3 Results

### 3.1 CO release from CORM-3 is transient and adversely affected by biological growth media

The conventional assay for CO release from CORMs is the change in absorbance of dithionite-reduced myoglobin (Mb) on binding CO [25]. Dithionite is a strong reducing agent and generates a much more strongly reducing environment than would normally be found inside cells. In order to test whether the presence of dithionite is critical in CO-release, we used an alternative assay by measuring CO gas in the headspace of reaction solutions by gas-phase Fouriertransform Infrared (FTIR) spectroscopy (Fig. 1b). Adding 100 μM CORM-3 to KPi buffer or various growth media at pH 7 without dithionite gave no detectable CO release within 30 min, consistent with previous observations [25], [31] and only low levels of CO (≤ 1.5 μM) were detected in the headspace after 10 h (data not shown). However, CO was released upon addition of 100 μM CORM-3 to KPi buffer or various bacterial or mammalian growth media in a sealed vessel that had been pre-reduced by adding 200 μM sodium dithionite prior to CORM-addition (the 0 min condition, Fig. 1c). The maximum yield of CO obtained was 50 μM, corresponding to ~ 0.5 mol CO per mol of CORM-3 (Fig. 1c), within the range observed previously for CO capture by red-Mb (0.5–0.7 mol CO per mol of CORM-3) [25] and confirmed here using the Mb assay and a 10-fold excess of dithionite (Fig. S1) [25]. Note that previously, no CO-release was detected by gas chromatography for CORM-3 dissolved in water at pH 4 or 5.5 over 2 h, but 0.19–0.2 equivalents of CO_2_ were detected [32].

CORM-3 prepared in water is relatively stable but in physiological media, such as Krebs-Henseleit buffer, it fails to convert red-Mb to the CO-Mb adduct, previously attributed to the medium “favoring the release of CO”, i.e. the CO was released prior to red-Mb addition [20], [33]. Incubation of CORM-3 in commonly used bacterial and mammalian growth media for only 5 or 10 min prior to dithionite addition led to a rapid decline in CO detectable in the headspace by FTIR (Fig. 1c). Furthermore, phosphate concentration (4 mM and higher) is a key determinant in reducing CO yield from ~ 0.5 mol to 0.1 CO per mol of CORM-3 (Fig. S1b) Thus, after only 10 min following addition of CORM-3 to solutions of standard buffers and media, less than 3% of the total CO ligands are biologically available. Because the antimicrobial properties of CORM-3 (inhibition of respiration rates or loss of cell viability) extend over 2–3 h [13], [31], and since CO is non-toxic [34], we conclude that toxicity cannot generally be attributed to CO release.

### 3.2 CORM-3 undergoes extensive ligand exchange

**Fig. 2:**
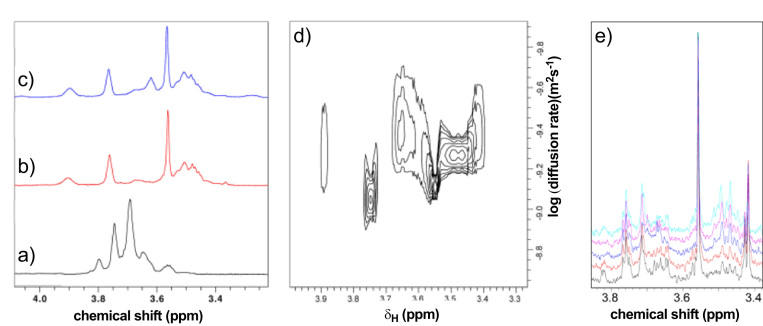
CORM-3 structural changes in phosphate-containing buffers. (a-c) ^1^H NMR spectra of CORM-3 (7 mM) immediately after dissolution in: (a) unbuffered distilled H_2_O pH 2.5; (b) 30 mM KPi buffer and adjustment to pH 7.4; and (c) adjustment of CORM-3 in H_2_O to pH 7.1 by addition of NaOH/HCl. The most intense peak in each spectrum corresponds to free glycine. (d) 2D diffusion-ordered spectroscopy (DOSY) spectrum of CORM-3 in KPi buffer pH 7.4. The horizontal axis is ^1^H chemical shift (ppm) and the vertical axis is the diffusion rate (log m^2^ s^−1^). The signal at 3.56 ppm corresponds to free glycine that is no longer bound to the Ru(II) of CORM-3. The other singlet signals correspond to the glycinate ligand of CORM-3, monodentate to the Ru(II) ion. The multiplet structure at 3.47 ppm corresponds to an asymmetrical glycine, most likely bidendate to the Ru(II) ion of CORM-3. (e) Titration of 0.1 mM CORM-3 with increasing concentrations of KPi pH 7.4 (bottom to top) 0.4, 1, 5, 10 and 30 mM. The most intense signals arise from free glycine (3.56 ppm) and mixtures of monodentate unsymmetrical glycinate species at 3.44–3.52 ppm and 3.66 – 3.68 ppm that are in reversible equilibria.

As CO release from CORM-3 declined rapidly following addition of the CORM to 30 mM KPi buffer at pH 7.4, we used ^1^H NMR to study structural changes in CORM-3 solutions, specifically ligand-exchange reactions. The only ^1^H signals in CORM-3 are from the glycinate Hα, which forms a four-line AB quartet, being magnetically non-equivalent (Fig. 1a). On dissolution of CORM-3 in water, the pH decreases to around 2.5, and the glycinate protons show the expected AB quartet, with an additional singlet at 3.69 ppm (Fig. 2a), matching the chemical shift of free glycine at this pH, suggesting that a significant fraction of the glycine detaches in water (Fig. S2). Upon adjustment of the pH to 7.1 the solution became yellow, which may indicate a further change of ligand. The ^1^H NMR spectrum of CORM-3 in H_2_O at pH 7.1 is altered in comparison to the spectrum at pH 2.5 (Fig. 2b).

The most intense signal in all spectra corresponds to free glycine, as indicated by its having an identical chemical shift and identical diffusion coefficient to free glycine, as measured by a 2D DOSY (Diffusion-Ordered Spectroscopy) experiment (Fig. 2d). The range of diffusion coefficients (Fig. 2d) indicates that the solution contains at least free glycine, two monodentate glycinate complexes, and two bidentate glycinate complexes. Next, the ^1^H NMR spectrum of CORM-3 in 30 mM KPi buffer after the adjustment of the pH to 7.4 was examined (Fig. 2c). This solution was also yellow. The spectrum resembles that of CORM-3 in H_2_O at neutral pH but contains additional signals.

Titration with different phosphate concentrations (Fig. 2e) demonstrates changes in the intensities of several bidentate glycinate species, indicating a complex mixture of species, including displacement of one or more non-glycine ligands by phosphate. This explains the reduced CO release in phosphate buffer (Fig. 1b and Fig. S1b), and implies that ligation of phosphate reduces CO release. The complexity of the spectra in Fig. 2 suggests further ligand exchange reactions, one of which is presumably the well-characterised WGSR [9], [35], in which hydroxide ions carry out nucleophilic attack on the CO ligand to produce a bound carboxylate. This would explain the dramatic lowering of pH on dissolution of CORM-3 in water, and why CO release is reduced substantially in buffer, by concomitant reduction in the number of CO ligands, and increased back-donation to any remaining CO ligands.

In summary, the speciation of CORM-3 in KPi buffer and neutral pH is complicated with at least six chemically distinct species, with different CO availabilities. Thus, structural changes generate a mixture of Ru-carbonyl and Ru-carboxylate species that are no longer able to release CO upon dithionite addition.

### 3.3 The toxicity of CORM-3 towards *E. coli* depends on composition of the growth media

It is striking that metal-carbonyl CORMs, particularly the Ru-based compounds, but not the non-metal boranocarbonate CORM-A1, are effective antimicrobial agents [31]. Since CO gas is tolerated at concentrations close to saturation (c. 1 mM) by *E. coli* [34] and considering that additional reactants (e.g. a CO acceptor such as Mb or a ligand to occupy the vacant coordination site on the metal ion) are needed to promote CO release from CORM-3 and CORM-2, we propose that the toxicity of Ru-based CORMs is due, not to CO, but to Ru(II) interactions with cellular components. Indeed, the potency of CORM-3 is abrogated in the presence of rich growth media: for example, 0.5–100 μM CORM-3 was sufficient to kill *P. aeruginosa* or *E. coli* cultures in chemically defined growth media [11], [13]. In contrast, ~ 400–1500 μM CORM-3 was required for activity against *E. coli, S. aureus, Helicobacter pylori* or *C. jejuni* cells grown in rich nutrient broths such as LB, Mueller-Hinton (MH) or Brain-Heart Infusion (BHI) [36], [37], [38]. Thus components in rich growth media may sequester Ru(II) and abrogate its toxic effects, as demonstrated for other metal ions [39]. This hypothesis was tested by following the effects of different media on CORM-3 toxicity.

**Fig. 3:**
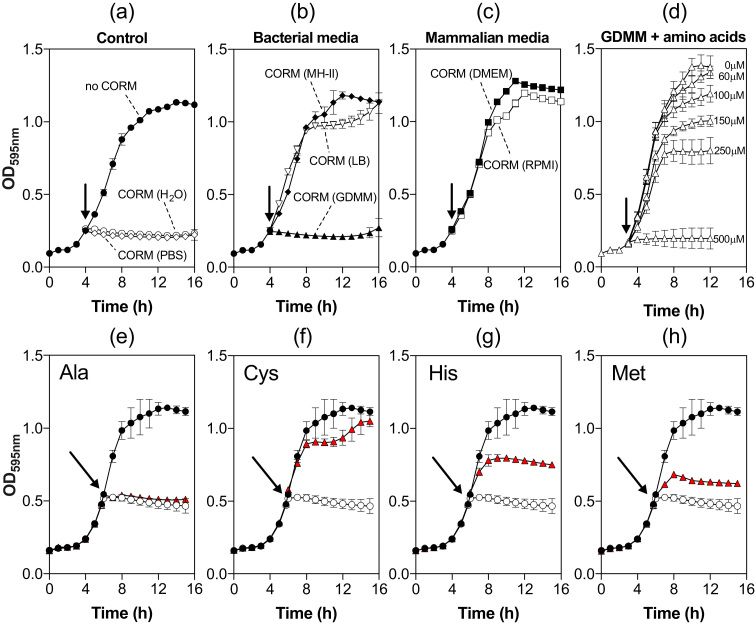
CORM-3 toxicity towards *E. coli* is alleviated by amino acid components of complex growth media. (a-c) CORM-3 stocks were mixed with a 10-fold excess of: (a) water or PBS pH 7.4; (b) bacterial media (MH-II, LB, GDMM); or (c) mammalian cell culture media (DMEM, RPMI-1640). These stocks were then added to *E. coli* cell cultures grown in GDMM to a concentration of 60 μM CORM (arrows). CORM-3 toxicity was determined by monitoring cell culture growth thereafter. A minus CORM control growth is shown in (a) for comparison. (d) The influence of amino acids on CORM-3 toxicity against *E. coli* was examined by supplementation of GDMM with 0.25% (w/v) casamino acids prior to the addition of 60–500 μM CORM-3 (arrow). Only 500 μM CORM-3 caused total growth inhibition. (e-h) Exogenous Cys, His or Met protect *E. coli* from the growth inhibitory effects of CORM-3. *E. coli* cultures were grown on GDMM and then 60 μM CORM-3 alone (black line, closed circles) or CORM-3 that had been pre-mixed for 10 min with a 2-fold excess of amino acid (red symbols) were added to the cultures (arrow). A no-reagent growth curve is shown for comparison (black line, closed circles). Control growths with 120 μM amino acids had no stimulatory or inhibitory effects and are omitted for clarity. All data are representative of ≥ 3 biological repeats, error bars represent ± standard deviation (SD). (For interpretation of the references to color in this figure legend, the reader is referred to the web version of this article.).

*E. coli* MG1655 cells were grown in minimal medium (GDMM), in which 60 μM CORM-3 completely inhibits growth and kills bacteria (Fig. S3). Prior to addition to cultures, CORM-3 stocks were pre-mixed into water (positive control; Fig. 3a), PBS (Fig. 3a), bacterial growth media (Fig. 3b), or mammalian cell culture media (Fig. 3c). Pre-mixing CORM-3 with simple phosphate salt-based media (PBS, GDMM) had no significant effect on CORM-3 toxicity to *E. coli* (Fig. 3a-b), but pre-mixing with rich bacterial media (LB or MH-II) (Fig. 3b) or commonly used mammalian cell culture media (DMEM or RPMI) (Fig. 3c)) completely abrogated growth inhibition. The final concentration of each medium used was < 5% (v/v) of the total culture volume and was without significant effect on culture growth (not shown). Furthermore, the Minimal Inhibitory Concentrations (MICs) for CORM-3 against *E. coli* were strikingly different in simple and rich media, for example, 4 μg mL^−1^ in GDMM but > 512 μg mL^−1^ in MH-II.

We note that the bacterial growth media that reduce CORM-3 toxicity the most (LB and MH-II; Fig. 3b) are those with least effect on CO release. (For interpretation of the references to color in this figure legend, the reader is referred to the web version of this article.) (Fig. 1b), while media compositions that provide the most effective antimicrobial activity (GDMM, phosphate buffers) have the greatest reduction in CO release (Fig. 1b), suggesting that the biological effects of CORM-3 are unrelated to CO release.

Amino acids and peptides are major components of rich biological growth media but are absent from defined minimal media. To investigate whether amino acids in rich media are responsible for the observed protective effects, CORM-3 was added to *E. coli* cells grown on GDMM supplemented with 0.25% (w/v) casamino acids (a casein hydrolysate); 500 μM CORM-3 was required to elicit the same growth inhibitory effects as 60 μM CORM-3 in the absence of amino acids (Fig. 3d). Thus, the amino acid components of rich growth media are identified as prime candidates responsible for the alleviation of growth inhibition by rich media.

### 3.4 The cellular toxicity of CORM-3 towards cultured mammalian cells is similarly dependent on composition of the growth media

**Fig. 4:**
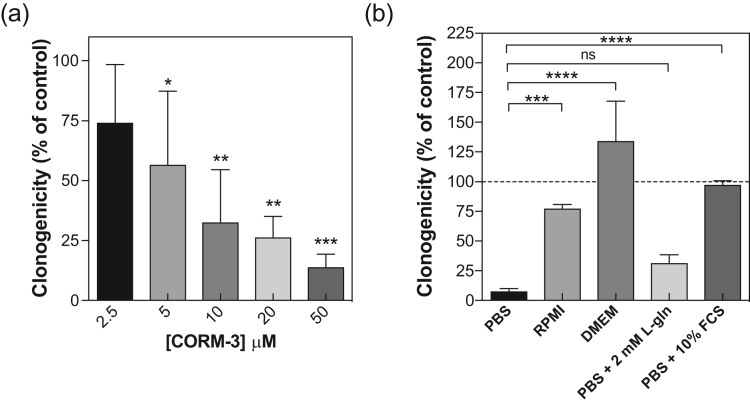
CORM-3 cytotoxicity towards mammalian cells is alleviated by components of cell culture media. (a) Percentage cell survival (clonogenicity) of human RKO cells following 1 h exposure to 2.5–50 μM CORM-3 in PBS. Clonogenicity was determined relative to a minus CORM control sample (* = p ≤ 0.05, ** = p ≤ 0.01, *** = p ≤ 0.001). Concentrations in excess of 50 μM CORM-3 resulted in a total loss of cell survival (data not shown). (b) Percentage survival of RKO cells after 1 h exposure to 25 μM CORM-3 in the presence of RPMI-1640, DMEM or PBS +/- media supplements. With the exception of L-Gln, addition of growth medium/supplements significantly protected cells against CORM-3 cytotoxicity or enhanced cell survival (*** = p ≤ 0.001, **** = p ≤ 0.0001). Data in (a-b) represent 3 biological repeats ± SD, significant differences were assessed via a one-way ANOVA followed by a Tukey's multiple comparisons analysis.

CORM-3 is reported to be non-toxic to mammalian cells. Clark [20] reported (but did not show) a lack of toxicity of 10–50 μM CORM-3 to rat H9c2 cardiac cells cultured in amino acid-rich DMEM, while Seixas [35] reported IC_50_ values > 100 μM CORM-3 for RAW264.7 cells, also in DMEM. Nobre reported minimal toxicity for a range of cultured eukaryotic cells, again in complex media such as DMEM [36] at up to 500 μM CORM-3. We therefore re-examined, in amino-acid free medium, CORM-3 cytotoxicity using the human colon carcinoma cell line RKO (ATCC CRL-2577), selected because the human gastrointestinal tract is likely to encounter pathogenic enterobacteria. Acute exposure to 2.5–50 μM CORM-3 for 1 h in PBS significantly decreased RKO cell clonogenicity relative to untreated control cells (Fig. 4a). Strikingly, treatment with 50 μM CORM-3 decreased RKO cell clonogenicity by 86% (p ≤ 0.001) and higher concentrations (75–500 μM) resulted in a total loss of clonogenicity (data not shown).

To identify protective components, RKO survival assays were performed in commonly used growth media or in PBS + supplements (2 mM L-Gln or 10% (w/v) Fetal Calf Serum (FCS)) (Fig. 4b). Treatment with 25 μM CORM-3 for 1 h in PBS decreased survival relative to untreated RKO cells in PBS by 92% (Fig. 4b), compared to only 23% in RPMI-1640 growth medium (Fig. 4b), whilst in DMEM the survival rate was enhanced relative to untreated controls (Fig. 4b). Supplementing PBS with 2 mM L-Gln was without effect (Fig. 4b), but PBS with 10% (v/v) FCS completely alleviated the cytotoxic effects of 25 μM CORM-3 (Fig. 4b). Thus, RPMI-1640, DMEM and/or 10% (v/v) FCS partially or fully alleviate the acute cytotoxic effects of CORM-3 against human RKO cells in vitro.

In summary, the effects of CORM-3 on cell growth are similar for bacterial and mammalian cells: it prevents growth in minimal media, but has no effect in complex media, due to their amino acid content. Thus, one of the main reasons for research interest in CORMs – their potential to kill bacterial but not mammalian cells – requires re-evaluation.

### 3.5 Exogenous Cys/thiols are the main components that alleviate the antimicrobial activities of CORM-3

To identify the amino acids responsible for protecting cells from CORM-3-induced toxicity, a growth inhibition screen was conducted: CORM-3 was mixed with a 2-fold excess of each individual amino acid for 10 min prior to addition to *E. coli* cultures. Most amino acids failed to prevent inhibition of growth by 60 μM CORM-3 (e.g. Ala, Fig. 3e, see Fig. S4 for the full amino acid screen) with three important exceptions. No inhibition by CORM-3 was observed in the presence of exogenous Cys, (Fig. 3f) as we reported before [11] and only partial growth inhibition was observed in the presence of exogenous His or Met (Fig. 3g, h). As the addition of these amino acids (120 μM) without CORM had no significant impact on growth (not shown), it is suggested that the extracellular sequestering of the Ru(II) of CORM by the exogenous amino acid prevents CORM toxicity. Other sulfur compounds, namely NAC, cystine (dicysteine), GSH, GSSG and sodium hydrosulfide (Fig. S5), protected *E. coli* cells from the growth-inhibitory effects of CORM-3. We previously attributed the effect of NAC to interference with the interaction of CO with respiratory cytochromes [11], but reactivity with the thiol now appears more likely.

**Fig. 5:**
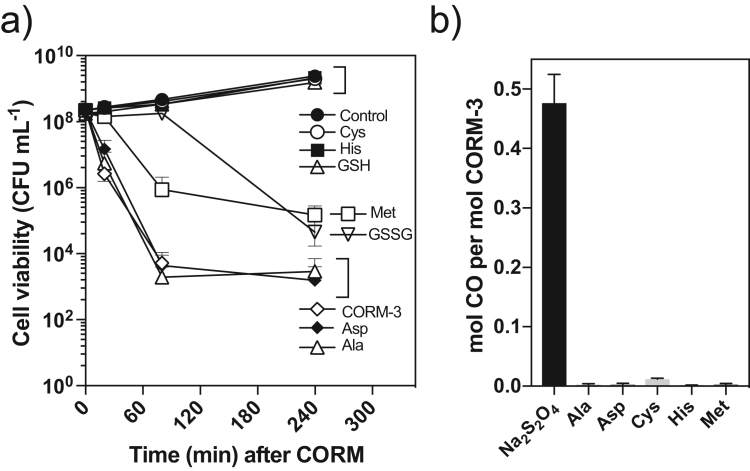
(a) Impact of exogenous Ala, Asp, Cys, His, Met, GSH or GSSG on CORM-3 bactericidal activity. *E. coli* cell cultures were grown on GDMM and then 60 μM CORM-3 alone or 60 μM CORM-3 that had been pre-mixed with a 2-fold excess of amino acid or glutathione was added (0 min). Samples for culture viability (CFU mL^−1^) were taken at time intervals thereafter for comparison with a no-CORM control. A 2-fold excess of Cys, His, Met or GSH was sufficient to fully alleviate CORM-3 toxicity, whereas Met or GSSG had a partially protective effect. (b) Impact of a 2-fold excess of Ala, Asp, Cys, His or Met on the level of CO released per mol CORM-3 as measured by gas-phase FTIR. A 2-fold excess of amino acid did not trigger significant levels of CO release. Data shown are the means of 3 biological repeats (a) and 3 technical repeats (b). Error bars represent ± SD.

As well as effects on growth, the bactericidal activity of CORM-3 was similarly affected by the same amino acids or glutathione (Fig. 5a). Pre-incubation of CORM-3 with a 2-fold excess of Cys, His or GSH restored culture viability to that of the no-reagent control, but Met or GSSG only partially protected viability (Fig. 5a). Asp or Ala had no effect (Fig. 5a). The alleviation of CORM-induced growth stasis or bacterial killing is unrelated to CO loss as no significant level of CO into the headspace of solutions was liberated by any amino acid unless 200 μM sodium dithionite was present (control) (Fig. 5b) and thus, CORM-3 interactions with selected amino acids do not elicit CO release. It has been previously reported that GSH/GSSG do not trigger CO release from CORM-3 [25].

The observation that addition of Cys or GSH to the medium abrogates CORM-induced antimicrobial effects immediately suggests that thiols (–SH) (and to a lesser extent, His, Met and GSSG) act by binding directly to Ru(II) and sequester it, since thiols are well characterised metal ligands. We next directly measured the binding of selected amino acids to CORM-3 using NMR.

### 3.6 Binding affinities of CORM-3 to selected amino acid residues parallel antimicrobial effects

**Fig. 6:**
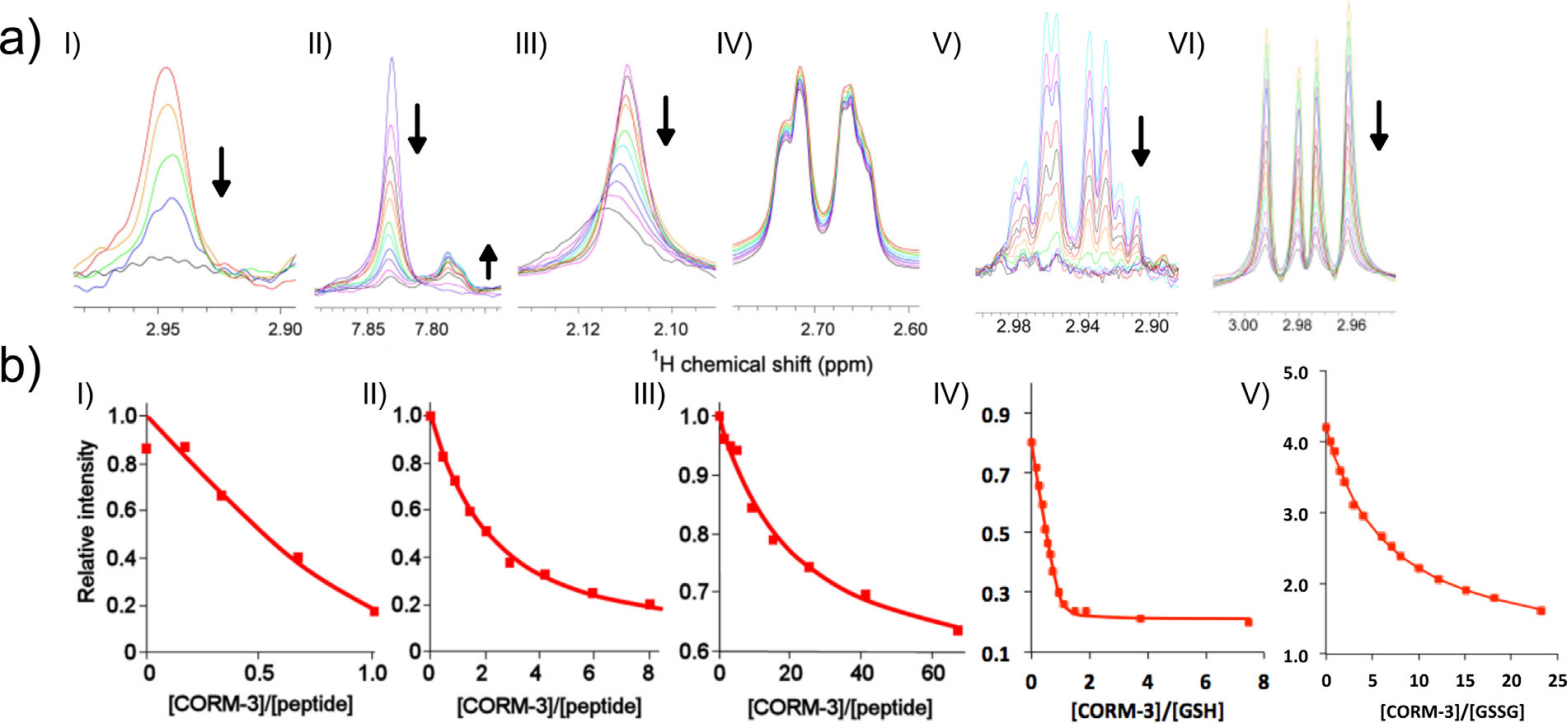
Binding affinities of CORM-3 to selected amino acids or glutathione as determined by ^1^H NMR. (a) ^1^H NMR spectra of: (I) Cys Hβ of A_3_CA_3_ peptide titrated with 0, 0.17, 0.33, 0.67 and 1.0 equivalents CORM-3; (II) His aromatic proton region of A_3_HA_3_ with 0, 0.43, 0.86, 1.4, 2.0, 2.9, 4.2, 5.9, 8.1, 11.5 and 18 equivalents CORM-3; (III) Met Hε region of A_3_MA_3_ with 0, 1, 3, 5, 9, 15, 24, 41, 67 and 134 equivalents CORM-3; (IV) Asp Hβ region of A_3_DA_3_ with 0, 0.33, 1.0, 1.5, 3.2, 6.5, 13 and 20 equivalents CORM-3; (V) GSH titrated with 0, 0.25, 0.375, 0.5, 0.625, 0.75, 0.875, 1.0, 1.25, 1.5, 2.0, 2.5 and 5.0 equivalents CORM-3; and (VI) GSSG titrated with 0, 0.5, 1, 1.5, 2, 3, 4, 6, 7, 8, 10, 12, 15, 18 and 23 equivalents CORM-3. Titrations were performed in 30 mM KPi buffer, with pH adjusted to ~ 7 – 7.6 where required. Arrows indicate the direction of signal intensities following increasing addition of CORM-3. (b) Binding curves of the decrease in intensity of ^1^H NMR signals in (a) corresponding to: Cys (I), His (II), Met (III), GSH (IV) and GSSG (V) upon increasing additions of CORM-3. 3. The estimated *K*_d_ of CORM-3 to each peptide was determined to be: 5 ± 3 μM for Cys, 800 ± 100 μM for His, 4700 μM for Met, 4 ± 2 μM for GSH and 9190 ± 1000 μM for GSSG.

Terminally blocked peptides A_3_XA_3_ were synthesized, composed of 6 Ala residues (A) with a central amino acid of interest, where X = Ala, Cys, His, Met or Asp. The only potential interaction with CORM-3 is expected to be the X sidechain. Peptides were titrated with increasing amounts of CORM-3 (Fig. 6), and binding interactions were measured by ^1^H NMR [27]. Upon titration of A_3_CA_3_ with CORM-3, the Cys Hβ proton signals decreased in intensity (Fig. 6aI). Fitting of the saturation curve indicated very strong 1:1 binding with a K_d_ of 5 ± 3 μM (Fig. 6bI). As more CORM-3 was added, additional signals were observed in the spectrum (Fig. 6aI), indicating slow exchange between free and bound forms, as expected from the strong affinity. Similarly, titration of A_3_HA_3_ with CORM-3 led to decreased intensities of the signals corresponding to the His ring, and increases in several new signals (Fig. 6aII), implying several bound species, as expected from the complex ligand exchange chemistry described above. Fitting of the saturation curve gave an estimated K_d_ of 800 ± 100 μM (Fig. 6bII), considerably weaker than the affinity of CORM-3 for the Cys-containing peptide. Titration of A_3_MA_3_ with CORM-3 exhibited a shift change and signal broadening of the Met Hε proton signal (Fig. 6aIII), indicative of an intermediate exchange rate, with an estimated K_d_ of ~ 4700 μM (Fig. 6bIII). Titration of A_3_DA_3_ (Fig. 6aIV) or A_7_ (Fig. S6) elicited no detectable spectral changes up to 20 or 134 equivalents CORM-3, respectively. Thus, the only detectable binding of amino acid sidechains to CORM-3 was Cys >> His > Met. These results exactly parallel the effects of these amino acids on cell growth (Fig. 3) and bactericidal activity (Fig. 5a), strongly implying that these amino acids in media reduce the antimicrobial activity of CORM-3 because of their binding to Ru(II).

In the next section, we present evidence that the antimicrobial activity of CORM-3 may be ascribable to its binding of intracellular thiols. It is therefore relevant to note that the major intracellular thiol, GSH, behaves as expected, binding strongly to CORM-3 (Fig. 6aV) with an affinity of 4 ± 2 µM and a 1:1 stoichiometry (Fig. 6bIV), whereas the oxidised form bound much more weakly (Fig. 6aVI), with an affinity of 9000 ± 1000 µM (Fig. 6bV)).

### 3.7 The cytotoxicity of CORM-3 strongly correlates with the intracellular accumulation of Ru

**Fig. 7:**
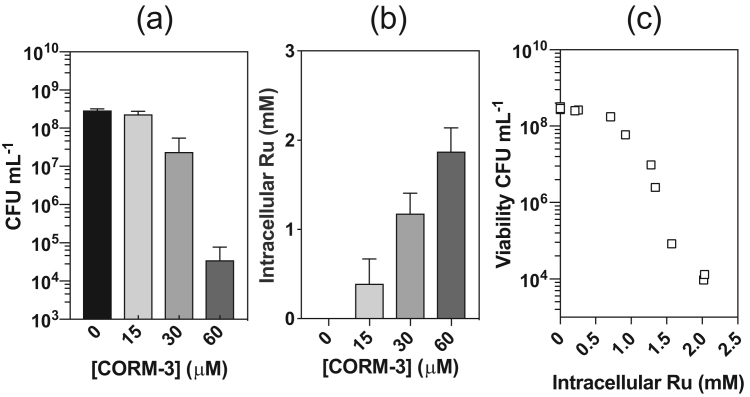
Viability of *E. coli* cell cultures (CFU mL^−1^) following 1 h exposure to 0–60 μM CORM-3 (a) was measured alongside cellular Ru levels by ICP-AES (b). In (c) the data in (a) and (b) are presented as CFU mL^−1^ vs. cellular Ru (mM) in each biological sample. There exists a strong negative correlation between the culture viability and the extent of cellular Ru accumulation (r^2^ = 0.877). Data are representative of 3 biological repeats and in (c) were assessed via Pearson's (two-tailed) correlational analysis (p ≤ 0.0001). Error bars represent ± SD.

We have demonstrated that the cytotoxic effects of CORM-3 are similar for both bacterial and mammalian cells, are not due to CO release, and are markedly reduced by presence of thiols and amino acids that bind directly to the Ru(II). To test the hypothesis that this protection is due to sequestering the Ru(II) extracellularly, we sought to measure intracellular Ru(II) accumulation by ICP-AES. CORM-3 (15–60 μM) caused a dose-dependent decrease in viability of *E. coli* cell cultures relative to untreated controls (Fig. 7a) and a concomitant increase in the intracellular Ru levels after 1 h exposure (Fig. 7b). There was a strong negative correlation (Fig. 7c) between culture viability and the corresponding level of intracellular Ru for CORM-3 (p ≤ 0.0001). Thus, the extent of bacterial cell killing by CORM-3 is directly and strongly associated with the extent of Ru accumulation by *E. coli* cells.

**Fig. 8:**
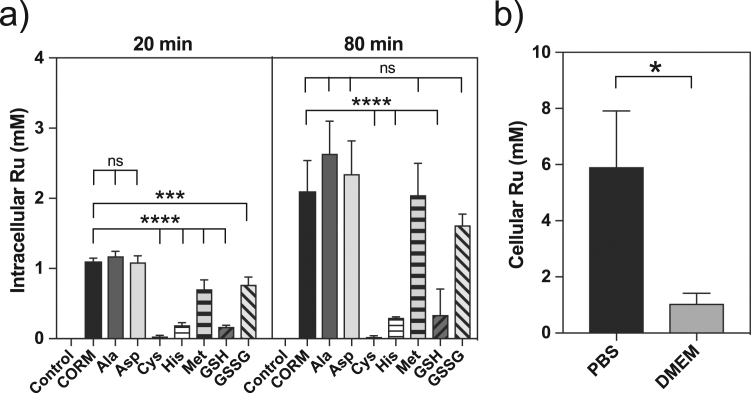
(a) Exogenous amino acids and glutathione influence CORM-derived Ru accumulation in *E. coli*. The level of cellular Ru of *E. coli* cells grown on GDMM was determined by ICP-AES after 20 and 80 min incubation with 60 μM CORM-3 alone (black bars) or 60 μM CORM-3 that had been pre-mixed with a 2-fold excess of Ala, Asp, Cys, His, Met, GSH or GSSG. Cys, His and GSH significantly reduced cellular Ru accumulation (p ≤ 0.0001). Met and GSSG slowed Ru accumulation with a significant reduction of cellular Ru at 20 min (p ≤ 0.001) but not at 80 min. Asp and Ala had no effect on Ru accumulation. (b) The level of cellular CORM-derived Ru accumulation by human RKO cells following 1 h exposure to 50 μM CORM-3 in either PBS (black bars) or DMEM (grey bars) was determined by ICP-AES. RKO cells incubated in PBS accumulated significantly higher levels of CORM-derived Ru than those treated in DMEM (p ≤ 0.05). Data represent 3 biological repeats ± SD. Significant differences were assessed via unpaired *t*-tests.

The level of CORM-derived Ru accumulated by bacteria following exposure to 60 μM CORM-3 alone was ~ 1.1 mM at 20 min and ~ 2.1 mM at 80 min (Fig. 8a). In contrast, pre-incubation of CORM-3 with a 2-fold excess of Cys, His or GSH led to dramatic reductions in Ru accumulation, particularly for Cys (Fig. 8a). Thus, the alleviation of CORM-3 antimicrobial effects by exogenous Cys, His or GSH is due to diminished intracellular Ru accumulation. Pre-incubation of CORM-3 with Asp or Ala had no significant effect on intracellular Ru (Fig. 8a), but Met or GSSG significantly lowered the level of cellular Ru accumulation after 20 min (Fig. 8a). Therefore, the partial protection of a 2-fold excess of exogenous Met or GSSG against CORM-3 antimicrobial effects correlates with diminished accumulation of CORM-derived Ru. The accumulation of CORM-3-derived Ru by human RKO cells followed the same pattern. The level of accumulated Ru by RKO cells following 1 h exposure to 50 μM CORM-3 was 5.9 ± 2.0 mM (Fig. 8b); in the rich medium DMEM, the level of CORM-3-derived Ru in RKO cells was significantly lower (Fig. 8b).

## 4 Discussion

CORM-3 is one of the most widely used CORMs in biological and medical research. Nevertheless, despite early classification of the compound as a ‘fast CO-releasing molecule’ [31], release of CO does not occur spontaneously in the media used for bacterial or mammalian cell growth. Indeed, CO was not detected previously in an aqueous solution of CORM-3 (or CORM-2) using either a CO-specific electrode or gas chromatography [31]. The inability of CORM-3 to transfer CO to deoxyhemoglobin in the absence of reducing agents correlates well with the absence of CO in the headspace of solutions of CORM-3 and other [Ru(CO)_3_Cl_2_(L)] (L=ligand) complexes [32], [35], [40]. Many of the biological effects of CORM-3 appear not to be due to CO liberation [8], [10], [16]. The toxic effects of CORM-3 are dramatically alleviated after pre-equilibration with Cys or GSH, and to a lesser extent His, Met or GSSG. Earlier, Desmard [11] suggested that inhibition of oxygen consumption by CORM-3 involved Cys residues of the respiratory chain, but we now demonstrate by NMR a direct interaction of exogenous Cys and other thiol compounds with the Ru(II) of CORM-3. With hindsight, a number of prior results have implicated a role of sulfhydryl compounds in attenuating the effects of CORM-3: our previous transcriptomic and respiration studies indirectly implicated sulfur metabolism as a factor in CORM-3 activities [16], and Nobre [41] noted that *E. coli met* mutants (defective in Met biosynthesis) showed increased sensitivity to CORM-2, suggesting that thiol-containing amino acids and peptides play a protective role.

Furthermore, (i) CO gas is an ineffective inhibitor of bacterial growth [34]; (ii) even a bacterial mutant that lacks all hemes (the classical biological target of CO) is sensitive to CORM-3 and indeed accumulates more Ru than wild-type cells [42]; (iii) a CORM-3 derivative that displays no CO release is toxic, reactive with sulfur compounds, and elicits complex gene transcription changes [16]; and (iv) Cys and GSH abolish the CORM-3-induced inhibition of bacterial membranes [16]. These findings substantiate the present view that CORM-3 toxicity is largely independent of CO and abrogated by sulfur species via reaction with the Ru center.

We note that the intracellular concentration of Ru is far greater than its extracellular concentration. This implies either an active transport mechanism, or (more likely) that, after the mixture of Ru complexes produced by dissolution of CORM-3 in media diffuses into cells, such complexes react with intracellular targets e.g. intracellular thiols. This would thus reduce the intracellular concentration of unbound Ru complex and thereby stimulate the influx of more Ru by mass action. The major intracellular thiol in *E. coli*, GSH, is present at millimolar concentrations in bacterial cells [43], [44]). We found that the MIC of CORM-3 against *E. coli* was lower against a GSH-deficient (*gshA*) mutant at 2 μg mL^−1^ compared to non-mutant cells at 4 μg mL^−1^. This suggests other targets for CORM-derived Ru and that intracellular thiols, such as GSH, may act as a buffer to protect Ru(II)-sensitive targets, such as active-site Cys or His residues on essential enzymes within bacterial cells. Obviously, in the absence of free Cys, glutathione, Met or His, CORM-3 can be toxic by combining with these residues in cell components, albeit without release of CO. Indeed, interactions with purified proteins in vitro have been reported: reactions of CORM-3 with hen egg white lysozyme and bovine serum albumin result in the formation of Ru(II)(CO)_2_ adducts on surface-exposed His residues, eliminating all other ligands to the Ru(II) ion [32], [45], [46]. A later study investigated the pharmacokinetics of a large panel of CO-releasing molecules containing Ru and group 6 metals (Cr, Mo, W), including CORM-3 (‘Complex 1’) [47]. Its IC_50_ was 63 mg/L (210 μM); many related complexes had lower toxicities. After administration to mice, the major proportion, assayed as Ru, remained in blood (predominantly protein-bound) after 10–60 min, and subsequent distribution and metabolism appeared slow. Thus, Ru binding to plasma proteins is a major factor in the pharmacodynamics of CORM-3.

The data presented here have important implications for the potential application of CORM-3 and similar Ru pro-drugs in therapeutic applications. Comparatively little attention has been paid to the toxicological profiles of these compounds or to the CO-depleted molecules in vivo. A number of toxic chemotherapies ascribe their mechanism of action to Ru, so that CO-depleted, but Ru-containing, CORM-2 or CORM-3 may also be detrimental to cellular functions [48], [49]. A detailed study [50] of the toxicology of CORM-2 and its ‘CO-depleted’ derivative (i-CORM-2) revealed significant cellular toxicity expressed as decreased cell viability, abnormal cytology, increased apoptosis and necrosis, cell cycle arrest and diminished mitochondrial activity. These treatment protocols (growth of cells, lactate dehydrogenase assays and cell adhesion assays) used complex medium formulations (DMEM with calf serum). Our results predict that, in simpler media, the observed toxicities would be much higher and in support we note that even 10 nM CORM-2 induces death in HL-1 heart cells [51]. Interestingly, inhibition of Complex I (NADH dehydrogenase) by CORM-2 or iCORM-3 was attributed, as here, to the reactivity of the Ru(II) ion [50]. An earlier study showed that CORM-2 reduced viability of vascular muscle cells after 24 h at 40, 170 and 210 μM CORM-2 (and higher concentrations) [52], but chronic applications are likely to require much longer contact times with host tissues and cells. However, cardiomyocytes and renal cells are sensitive to 20 μM CORM-2, a concentration advocated for therapeutic use [53], [54].

We conclude that in physiological conditions CORM-3 releases little CO. Nevertheless, very high concentrations of extracellular CORM-3 (e.g. 100 μM) release sufficient CO to access intracellular cytochromes (but present at < 0.1 μM in cells), even in the absence of dithionite [17]. Furthermore small increases in CO levels are observed in CORM-3-treated rodent organs after sacrifice [35] Our data suggest only low levels of CO release in biological conditions and so the numerous studies that have interpreted CORM-3 experiments as due to CO release require re-evaluation. Rather, on dissolution in buffer or media, CORM-3 undergoes a series of ligand displacement and modification reactions, to generate species with accessible Ru(II) centers. These species are transported and accumulated into bacterial and mammalian cells, where the Ru reacts with cellular components such as glutathione. One mechanism of cytotoxicity is therefore predicted to be perturbation of intracellular redox balance, depletion of glutathione-dependent systems and Ru(II) coordination to metal-sensitive targets as observed for toxicity of other non-essential metal ions^10^. Ru also reacts with intracellular Cys, His, and Met free in the cytosol in proteins. Much of the selective cytotoxicity against bacterial rather than mammalian cells is due to cysteine and other amino acids in mammalian growth media, which react with Ru in the media and prevent its intracellular uptake. It is the previously unappreciated balance between the opposing effects of toxicity of CORM-3 and its inactivation that has led to confusing, and even inconsistent, observations and conclusions in earlier studies.

## 5 Conclusions

Carbon monoxide-releasing molecules (CORMs) are widely used to deliver physiologically relevant levels of CO experimentally or therapeutically, but are also investigated as novel antimicrobial agents and as a contribution to the threats of antimicrobial resistance. However, the molecular basis of their toxicity is not well understood. In this study we showed that CORM-3 releases little CO under commonly used conditions, and that the active antimicrobial agent is Ru(II), which binds tightly to thiols. This explains why thiol-rich, complex media protect cells against CORM-3. Cysteine and reduced glutathione bind Ru(II) extremely tightly and are effective protectants. These results necessitate a major reappraisal of the biological effects of CORM-3 and related CORMs.
