# Supplementary material for: A thiol-reactive Ru(II) ion, not CO release, underlies the potent antimicrobial and cytotoxic properties of CO-releasing molecule-3
Source: Redox Biol. 2018 Jun 30;18:114–23. doi: 10.1016/j.redox.2018.06.008 (PMC6067063; doi:10.1016/j.redox.2018.06.008)
Supplement: Supplementary file 1 — Supplementary material [file mmc1.docx]

**Supporting Information for:**

**A Thiol-Reactive Ru(II) Ion, not CO Release, Underlies the Potent Antimicrobial and Cytotoxic Properties of CO-Releasing Molecule-3**

Hannah M. Southam^a^, Thomas W. Smith^b,^, Rhiannon L. Lyon^a^, Chunyan Liao^a^, Clare R. Trevitt^a^, Laurence A. Middlemiss^b^, Francesca L. Cox^b^, Jonathan A. Chapman^b^, Sherif F. El-Khamisy^a^, Michael Hippler^b^, Michael P. Williamson^a,1^, Peter J. F. Henderson^c,1^, Robert K. Poole^a,1,^*

*^a^ Department of Molecular Biology and Biotechnology, The University of Sheffield, Western Bank, Sheffield S10 2TN, UK*

*^b^ Department of Chemistry, University of Sheffield, Western Bank, Sheffield S3 7HF, UK*

*^c^School of Biomedical Sciences and Astbury Centre for Structural Molecular Biology, University of Leeds, Leeds, LS2 9JT, UK.*

^1^Corresponding authors

- Correspondence to Robert Poole, Department of Molecular Biology and Biotechnology, The University of Sheffield, Western Bank, Sheffield S10 2TN, UK

E-mail address: [r.poole@sheffield.ac.uk](mailto:r.poole@sheffield.ac.uk) (R. K. Poole)

**This PDF file includes:**

Supplementary Information Text (pg. 2)

Materials and Methods (pg. 2)

1. Measurements of bacterial growth and viability. (pg. 2)

2. Measurements of CO release from CORM-3 by Mb assays (pg. 2)

3. Determination of Ru content of *E. coli* cells by ICP-AES (pg. 2)

4. Routine growth of RKO cells (pg. 3)

5. Measurements of RKO cell survival (clonogenicity) (pg. 3)

6. Statistical analysis (pg. 3)

Supplementary Data - Figs. S1 (pg. 4) to S6 (pg. 9)

References for SI reference citations (pg. 10)

**Supplementary Information Text**

Materials and Methods

**1. Measurements of bacterial growth and viability.** Growth was monitored by measuring culture turbidity (optical density (OD)) at 600 nm in a Jenway 7305 spectrophotometer or in 96-well microtiter plates at 595 nm in a Tecan Sunrise^TM^ plate reader. Culture viability was determined via the viable counts method, which is based on the ability of a single viable *E. coli* cell to form a colony when seeded on solid medium. Briefly, 10-fold serial dilutions (10^-1^ – 10^-8^) of cell culture samples were prepared in sterile PBS and then 4 × 10 μL drops of each dilution were transferred to nutrient agar plates. Plates were incubated overnight at 37 °C to permit colony growth. Cell viability was determined as the average number of colony forming units (CFU) counted per mL of culture (CFU mL^-1^).

**2. Measurements of CO release from CORM-3 by myoglobin (Mb) assays.** Mb stocks were prepared by dissolution of Mb from equine skeletal muscle (Sigma-Aldrich) in PBS and were reduced by addition of a few granules of sodium dithionite. To this ferrous Mb solution (red-Mb, 11 – 17 μM), CORM-containing solutions were added to a final concentration of 10 μM. Samples were incubated at room temperature for 5 – 30 min and the level of CO-Mb was determined from a difference spectrum (CO-Mb *minus* red-Mb) at 400 – 700 nm in a Cary-50 spectrophotometer (Varian) relative to a red-Mb baseline. Concentrations of the CO-ligated globin were calculated using a molar extinction coefficient of 177 mM^-1^ cm^-1^ (~ 421 nm *minus* 440 nm) in the Soret region^1^. Saturated CO-Mb spectra were obtained by bubbling red-Mb with CO gas for 2 min.

**3. Determination of Ru content of *E. coli* cells by ICP-AES.** Samples of cell culture were harvested, washed thrice in 0.5 mL 0.5 % (v/v) cold nitric acid, then solubilised in Aristar nitric acid (69 % (w/v)) and placed in a sonicator bath for 30 min. The resulting digest was then analysed for Ru content via a Spectro CirosCCD (Spectro Analytical) inductively coupled plasma-atomic emission spectrophotometer (ICP-AES). Levels of Ru in the samples were determined by a calibration curve using multi-element standard solutions containing 0.1, 0.2, 5 and 10 mg L^-1^ Ru. The approximate amount of Ru per cell was determined via estimates of the total number of viable cells per dry weight (mg) of culture and literature estimates of *E. coli* cell volume of 0.86 μm^3^ (8.6 × 10^-16^ L)^2^.

**4. Routine growth of RKO cells.** RKO (ATCC CRL-2577) cells were routinely grown under strict aseptic conditions in RPMI-1640 growth medium supplemented with 10 % (v/v) fetal calf serum (FCS), 2 mM L-glutamine and Pen Strep (a mixture of penicillin G sodium salt and streptomycin sulfate, ThermoFisher; 50 μg/mL). Cell layers were grown in 25 – 75 cm^2^ canted neck, vented cap, sterile polystyrene cell culture flasks to 80 – 90 % confluence (surface coverage) prior to experiments or sub-culturing. Cell cultures were incubated at 37 °C in a humidified 5 % CO_2_ atmosphere.

**5. Measurements of RKO cell survival (clonogenicity).** RKO cells, grown to 80 – 90% confluence, were trypsinized and then centrifuged at 400 × *g* to remove residual growth medium. The cell pellet was gently resuspended in warm PBS and then re-centrifuged to remove the supernatant. Cell pellets were resuspended in PBS for counting on a hemocytometer slide and then further diluted in PBS to a final concentration of 10^6^ cell mL^-1^. Samples of cell suspension (1 ml) were added to sterile 20 mL tubes and freshly prepared CORM-3 was added (up to 500 μM). The lids of the tubes were loosely secured to prevent anoxia and suspensions incubated for 1 h at 37 °C. Then, suspensions were diluted to 5 × 10^5^ cells mL^-1^ by the addition of RPMI-1640 medium + supplements, and then further diluted with medium to 5-16 × 10^3^ cells mL^-1^. A sample (10 ml) of each dilution was transferred to sterile cell culture dishes in triplicate and then incubated for 9 days when colonies became visible. The medium was removed and the plates were allowed to air-dry for 5 min before fixing to the plate by dehydrating in 10 mL 80 % (v/v) ethanol for 15 min. The ethanol was removed and plates were left to air-dry for 30 min. Colonies were stained with 1 % (w/v) methylene blue for 1 h and then gently washed with H_2_O. Clonogenicity, (i.e. survival and the subsequent ability to form clones) of RKO cells was determined by the average number of colonies on the plate, where one colony is representative of one viable cell after acute exposure to the CORM ^3^. The % clonogenicity after CORM treatment was determined by enumerating CORM-treated RKO cells to RKO cells treated under the same conditions without CORM.

**6. Statistical analysis.** Determinations of means and standard deviations (SD) of data sets were conducted in Microsoft Excel. Statistical analysis (ANOVAs, Tukey’s multiple comparisons tests, t-tests, Pearson’s correlational analysis) and graph fitting for standard curves used GraphPad Prism software.

**Supplementary Data**

**Fig. S1**

Fig. S1. Total mol CO released per mol CORM-3 as determined via the commonly-used carbonmonoxy-myoglobin (CO-Mb) assay. (a) Representative CO-Mb difference spectrum after CORM-3 addition red-Mb (inset). (b) Yield of CO per mol of CORM-3 to red-Mb after 20 min incubation in various [KPi]/CORM-3 ratios.

**Supplementary Data**

**Fig. S2**

Fig. S2. . ^1^H-NMR spectra of CORM-3 (7 mM) in 30 mM KPi buffer pH 7.4 before (a) and after spiking with 5 mM glycine (b). The peaks corresponding to free glycine are indicated by the arrows.

**Supplementary Data**

**Fig. S3**

Fig. S3. CORM-3 inhibits cell growth and is bactericidal to *E. coli* MG1655 cell cultures grown on glucose defined minimal medium (GDMM). (a) Cell growth, assessed by monitoring culture optical density (OD_600_), in the absence of CORM-3 (closed circles) or after the addition of 15 μM (dashed line, closed squares), 30 μM (open squares), 60 μM (dashed line, closed triangles) or 120 μM (open triangles) CORM-3. Growth was completely inhibited at ≥ 30 μM CORM-3. (b) Culture viability, assessed via colony forming units per mL (CFU mL^-1^), in the absence of CORM-3 (black bars) or after the addition of 15 μM (white bars), 30 μM (light grey bars), 60 μM (dark grey bars) or 120 μM (black/grey bars) CORM-3. Arrows indicate where the culture viability was undetectable. CORM-3 caused a dose-dependent increase in cell killing, with a complete loss in culture viability at 120 min at ≥ 60 μM CORM-3. Data represent 3 biological repeats ± SD.

**Supplementary Data**

**Fig. S4**

Fig. S4. Exogenous Cys, His or Met protect *E. coli* from the growth inhibitory effects of CORM-3. *E. coli* cultures were grown to mid-exponential growth phase on GDMM and then 60 μM CORM-3 alone (black line, open circles) or CORM-3 that had been pre-mixed for 10 min with a 2-fold excess of amino acid (grey or red lines/symbols) were added to the cultures (arrow). A no-reagent growth curve is shown for comparison (black line, closed circles). Only Cys, His and Met had any effect on the growth-inhibitory effects of CORM-3 (highlighted in red). Control growths with 120 μM amino acids had no deleterious or advantageous effects on growth and so are omitted for clarity. Data shown are the means of 3 technical and 3 biological repeats. Error bars represent ± SD.

**Supplementary Data**

**Fig. S5**

Fig. S5. Exogenous sulfur compounds protect *E. coli* cells from CORM-3 induced growth inhibition. *E. coli* cell cultures were grown to early exponential phase on GDMM and then either 60 μM CORM-3 alone (black line, open circles) or 60 μM CORM-3 that had been pre-incubated with a 2-fold excess of sulfur compound (grey line, closed squares) was added as indicated by the arrow. A minus CORM control (black line, closed circles) is shown for comparison. Reduced thiol-containing compounds N-acetyl cysteine, reduced GSH and Na hydrosulfide had the most protective effect against CORM-3 induced growth inhibition. Oxidised thiols oxidised Cys and GSSG had only a partially protective effect against CORM-3 induced growth inhibition. Data represent means of ≥ 3 technical repeats and 2 biological repeats. Error bars represent ± SD.

**Supplementary Data**

**Fig. S6**

**Fig. S6.** Titration of A_7_ peptide with CORM-3 by ^1^H-NMR. Ala methyl Hβ region of the ^1^H-NMR spectrum of 4.5 μM A_7_ peptide titrated with CORM-3 in 30mM KPi at pH 7.4 – 7.6. Due to the low solubility of the peptide, the starting solution also contained 3.3 % (v/v) *d_6_*-DMSO. Titration of A_7_ with 0, 2, 8, 32, 64 and 134 equivalents CORM-3 did not decrease the intensity of the Ala Hβ signal (1.4 ppm) relative to the TSP standard, thus the binding of Ala to CORM-3 is undetectable by ^1^H-NMR. Other signals in the spectrum are CORM-3 (1.17 ppm) or from impurities.

**Supplementary Information Text References**

1. Wood, P. M., *Biochim. Biophys. Acta* **1984,** *768*, 293.

2. Nanninga, N., *Molecular Cytology of Escherichia coli*. Academic Press Inc. (London) Ltd.: London, 1985.

3. Franken, N. A.; Rodermond, H. M.; Stap, J.; Haveman, J.; van Bree, C., *Nat Protoc* **2006,** *1*, 2315.
